# Three-dimensional optic nerve head images using optical coherence tomography with a broad bandwidth, femtosecond, and mode-locked laser

**DOI:** 10.1007/s00417-014-2870-5

**Published:** 2014-12-12

**Authors:** Takuhei Shoji, Hiroto Kuroda, Masayuki Suzuki, Motoyoshi Baba, Makoto Araie, Shin Yoneya

**Affiliations:** 1 Department of Ophthalmology, Saitama Medical University, 38 Morohongo Moroyama-machi, Iruma, Saitama 350-0495 Japan; 2 Advanced Laser Medical Center, Department of Ophthalmology, Saitama Medical University, Iruma, Saitama Japan; 3 Department of Ophthalmology, Kanto Central Hospital, Tokyo, Japan

**Keywords:** OCT, Optic nerve head, Lamina cribrosa, Three-dimensional imaging

## Abstract

**Purpose:**

The aim of this study was to demonstrate the fine laminar structure of the optic nerve head (ONH), in vivo, using a broad wavelength, ultra-high resolution, and optically coherent tomography (OCT) system.

**Methods:**

This high-resolution OCT system, based on a 200 nm bandwidth spectrometer and an 8 femtosecond ultra-short, mode-locked, coherent laser light source, enabled in vivo cross-sectional ONH imaging with 2.0 μm axial resolution. A total of 300 optic disc B-scans, which consisted of 300 × 2048 pixels, were obtained in 10 μm steps. Three-dimensional images were rendered from these images to obtain n face images of the optic disc. Fundus photography, scanning laser ophthalmoscopy (SLO), and standard OCT were also performed for all subjects.

**Results:**

Thirty-six eyes of normal subjects and ten eyes of glaucoma patients with mean age of 40.0 ± 10.0 years were enrolled in this study. Sequential en face images, from the ONH surface to deeper layers, were reconstructed in 2.0 μm steps. Observation of the images indicated variations in the shape and arrangement of the lamina pores at different depths. Clear lamina pores were identified by this technique in 44 eyes, compared with the fundus camera (identified in six eyes), SLO (identified in 14 eyes), and standard OCT (identified in 24 eyes) (all comparisons, *p* < 0.001).

**Conclusions:**

The fine structure of the ONH could be resolved in vivo using our OCT, providing improved imaging that can be used in research and clinical applications for a better characterization of the anatomical and pathological features associated with glaucoma.

**Electronic supplementary material:**

The online version of this article (doi:10.1007/s00417-014-2870-5) contains supplementary material, which is available to authorized users.

## Introduction

The optic nerve head (ONH), in particular the lamina cribrosa (LC), is considered to be the primary site of axonal injury in glaucoma. Since 1947 when Wilczek reported that the axons of the retinal ganglion cells aggregate into bundles to pass through the laminar pores [1], structural changes in the LC have been implicated in the pathogenesis of glaucomatous optic neuropathy, and there is growing evidence that the laminar region is the principal site of the retinal ganglion cell (RGC) axonal insult in this disorder [2–4].

An early study reported that changes in the size and shape of the laminar pores correlated with progression of glaucoma [5]. Other studies reported that normal subjects showed approximately round lamina pores, whereas pores became more elongated and less circular with increasing field loss in glaucoma patients [3, 6]. These reports, however, studied only the surface structure of LC, because neither the fundus camera nor scanning laser ophthalmoscope (SLO) images could be obtained from the deep layer of the LC, in vivo. However, spectral/Fourier domain OCT uses a broadband light source and a spectrometer to measure the interference spectrum [7–9]. The improved speed has enabled three-dimensional (3D) and high-definition imaging of the retina, and has enhanced visualization of retinal diseases [10–13].

Although 3D imaging of deep ONH and LC is clinically relevant, a recent study reported that it was necessary to scan 42 times and reconstruct averaging images to improve visualization. It, therefore, required 10–20 minutes to scan all raster images using a standard commercial Spectral-domain(SD)-OCT [14].

Spatial resolution in the axial (depth) direction of OCT is largely determined by the coherence length of the light source, and high resolution and penetration OCT has been sought to obtain 3D optic nerve head images without averaging. Recently, we have developed a new OCT using a wide bandwidth, mode-locked (ML), femtosecond laser light source, which could obtain a higher resolution image than commercial standard SD-OCT instruments [15, 16]. The high-resolution image acquisition enabled a faster clearance of a single scanned image, resulting in far faster construction of 3D images than the standard commercial SD-OCT instruments currently available.

The purpose of the present study was therefore to investigate whether this wide-wavelength, high resolution SD-OCT improved the visualization of the laminar pore formation and the laminar layer.

## Methods

The Ethics Committee of Saitama Medical University approved this cross-sectional clinical trial, which was conducted in accordance with the tenets of the Declaration of Helsinki. Subjects were included only if they were at least 20 years old, fulfilled the eligibility requirements detailed below, and signed an informed consent form. Inclusion criteria for all participants were as follows: best-corrected visual acuity of 20/40 or better, healthy anterior segment appearance on slit-lamp biomicroscopy examination, open angles on gonioscopy, normal posterior segment appearance on indirect ophthalmoscopy, normal optic disc appearance on direct ophthalmoscopy under a dilated pupil, and if necessary, reliable and normal visual field (VF) results in accordance with the criteria of Anderson and Patella [17]. VF testing was performed with optical correction by contact lenses or by trial lenses by standard automated perimetry (SAP) using the Humphrey Visual Field Analyzer with the 30-2 Swedish interactive threshold algorithm standard (Carl Zeiss Meditec, Inc. Jena, Germany). Subjects were excluded if any evidence suggested a history of ocular surgery (except for uncomplicated cataract surgery), or other diseases affecting the VF (e.g., neuro-ophthalmological diseases, uveitis, retinal or choroidal diseases, and trauma).

## In vivo 3D OCT imaging

**Fig. 1:**
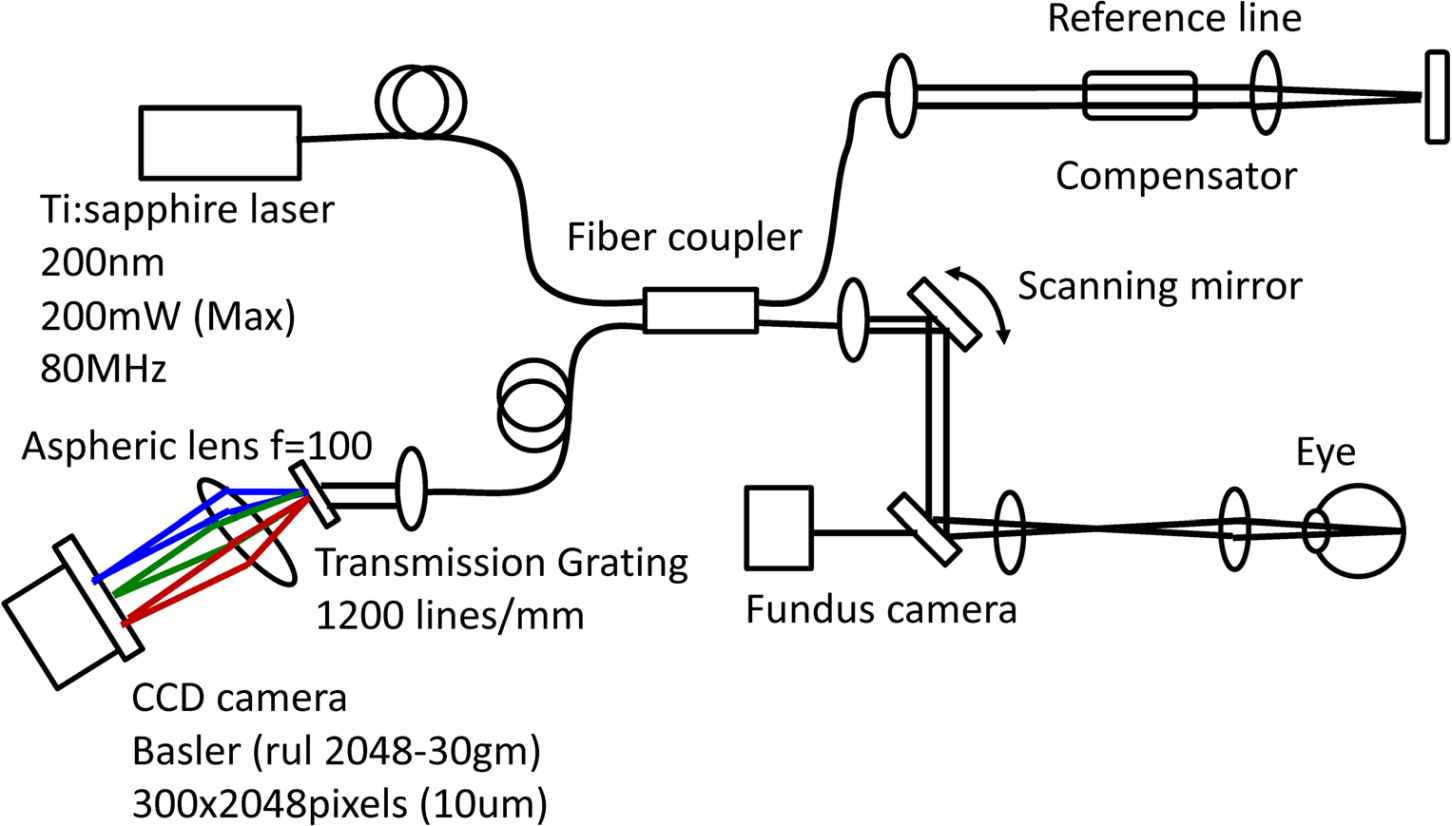
Schematic of optical coherence tomography (OCT) using a mode-locked (ML), ultra-short, femtosecond laser source and a wideband spectrometer

A schematic of the ultra-high resolution SD-OCT system is shown in Fig. 1. This OCT system was built by the Advanced Laser Medical Center (ALMC) at Saitama Medical University. The details of our SD-OCT system are described elsewhere [15, 16]. In brief, we developed this high-speed SD-OCT instrument using an ultra-broadband Kerr lens, ML Ti:Sapphire laser, and a wideband spectrometer. The spectral bandwidth of the light source was 200 nm full width at half maximum (FWHM) at a central wavelength of 840 nm. A high-speed CCD camera with 2048 × 300 pixels (Basler, Ahrensburg, Germany) was used as the detection system. The measurement speed was 50,000 A-scans/second, and measured depth resolution into the tissue was 2.0 μm. The interferometer was attached to a semi-custom made, fundus-scanning head system.

**Fig. 2:**
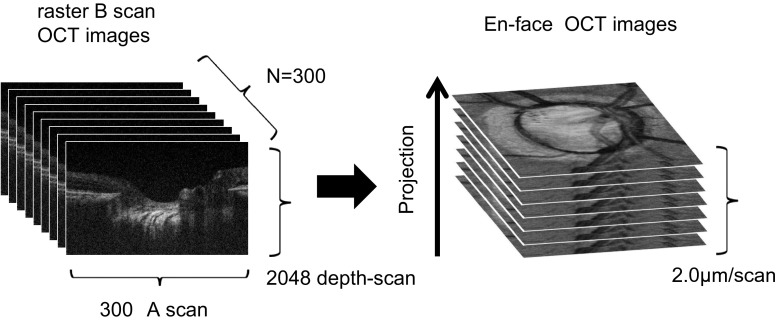
Image of an optic disc created from OCT

A raster scanning protocol with 300 B-scans with 300 A-scans (with 2048 pixels/A-Scan) covering a 3.0 × 3.0 mm square region centered at the ONH was used for volumetric scans. Volumetric rendering of the 3D-OCT data set was performed to reconstruct longitudinal and en face cross-sections using image processing software (Amira 5.4.3, Mercury Computer Systems Inc., Chelmsford, MA, USA). A fundus image was generated as an en face projection image from the 3D data by integrating the magnitudes of the OCT signals at each lateral position along the axial direction. The total data acquisition time for a single 3D-OCT (volumetric) image was 3.0 seconds without an eye tracking system. The optic disc was also imaged by both a commercial SLO (Spectralis; Heidelberg Engineering, Heidelberg, Germany) and a digital 30 degree fundus camera (Zeiss FF450, Carl Zeiss, Jena, Germany) on the same day. Figure 2 illustrates how the optic disc image was obtained using this OCT.

En face image acquisitions were obtained using OCT with a super luminescent diode (SLD), a commercial SD-OCT. The optic discs of the subjects were also evaluated by standard SD-OCT using SLD as the light source (Spectralis OCT) using the Enhanced Depth Imaging (EDI) method. Subjects were imaged through dilated pupils. A 5–15 degree rectangle for horizontal scans covering the optic disc was scanned with approximately 131 sections, which were 10 − 12 μm apart (the slicing distance was determined automatically by the machine). Scanning was performed by averaging two frames. The total data acquisition time for these images was 5 to 15 seconds (depends on subject’s cooperation). A 3D volumetric image was reconstructed from the B-scan images using the same image processing software (Amira 5.4.3), followed by generation of en face images.

The appearance of the laminar pore was independently evaluated by two readers (T.S. and M.S.) who were masked to all other data about the eyes. If the image identified the lamina pore and the pore margin, the image was defined as a clear lamina pore image, and if the image identified the lamina pore but did not identify the pore margin, the image was defined as an obscure lamina pore image. Images of each eye were displayed on a monitor. If the decisions of both examiners were not in agreement, consensus was reached by group review.

## Statistical analyses

Data were recorded as frequencies with percentages for categorical variables. When appropriate, either the chi square test or Fisher’s exact test was used for categorical variables. McNemar’s and Cochran’s Q tests were used to assess differences in proportions. A *p*-value less than 0.05 was defined as a statistically significant difference. All statistical analyses were performed using SPSS ver. 22 software (Japan IBM, Tokyo, Japan).

## Results

This study initially involved 24 subjects. Of these, one subject was excluded because of difficulty in cooperation during the image acquisition. A total of 23 subjects (46 eyes) were finally included for analysis. Thirty eyes (65 %) were male and ten eyes were glaucomatous. The average age was 40.0 ± 15.0 years (range, 24–73 years). Table 1 summarizes the baseline characteristics of the study subjects.

**Table 1:** Subjects’ clinical demographics

Variables	
Male (eyes, %)	30 (65.2)
Age (yrs)	40.0 ± 15.0
Spherical equivalent error (*D*)	−2.3 ± 2.7
Glaucoma patients (eyes, %)	10 (21.7)

Yrs, years; D, dioptres

Figure 2 shows an OCT fundus image obtained by intensity integration, together with single scans obtained in vivo. A 3D image was acquired that visualized deep layer connective tissue in the LC, with a multilaminar sheet structure. Video clip 1 (available online at supplemental files 1) shows raster B-scans and en face images as moving images.

## Observation of lamina beams within the LC

**Fig. 3:**
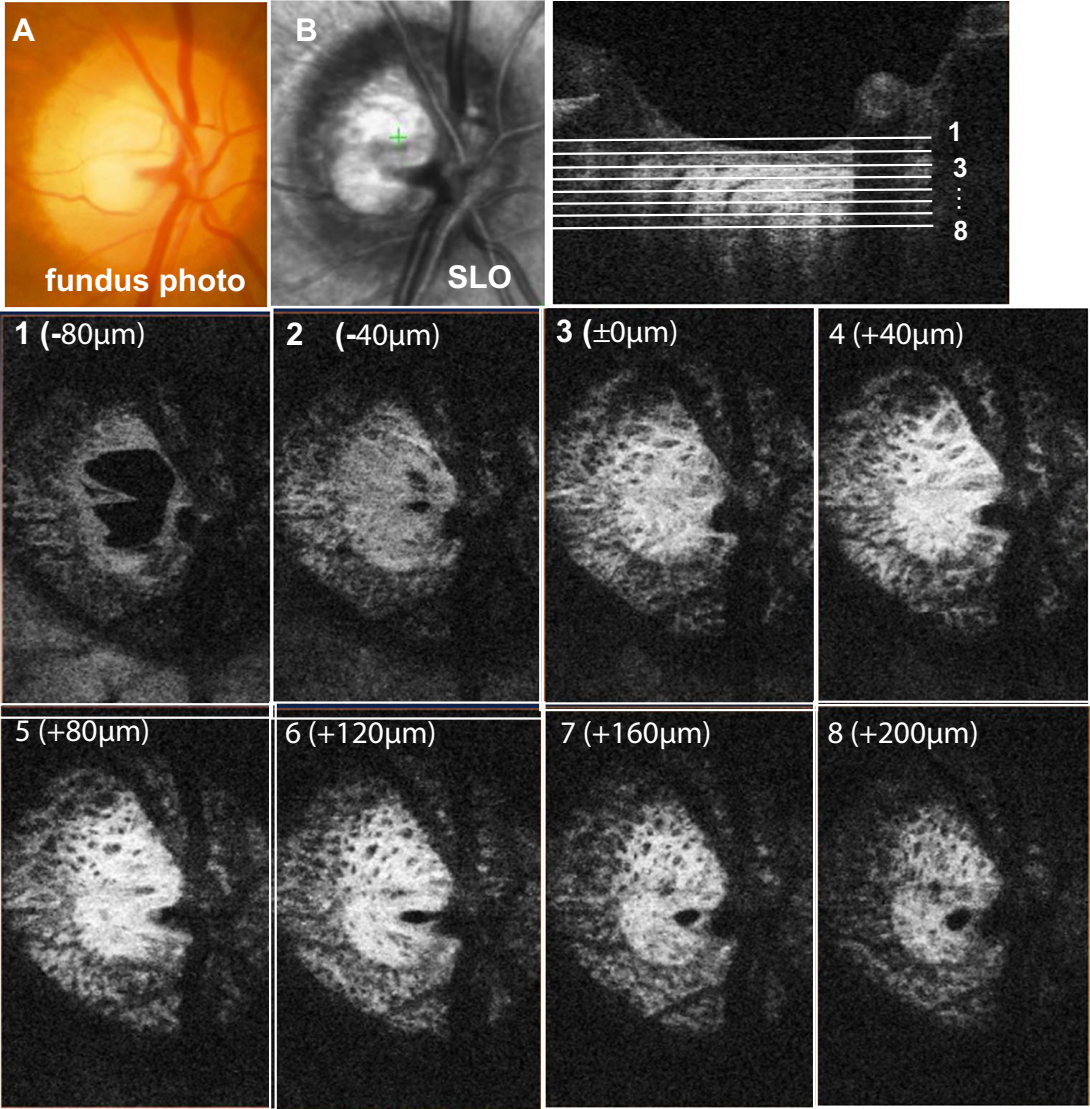
Visualization of the en face image of an optic nerve head using OCT, from the surface to the deep layer of the lamina cribrosa, compared with a fundus photograph and with scanning laser ophthalmoscopy (SLO). 1-8, Representative thin slice images of 2.0 μm axial depth, each with 40 μm steps, at different levels of the laminar cribrosa. Image 3 is located on the anterior surface of the lamina cribrosa

Figure 3 shows our OCT visualization of thin slice, en face images of an optic disc, from the surface to the deep layers of the LC, with comparison with the fundus photograph (3A) and with SLO (3B). Thin slice, en face images were obtained by 2.0 μm step scans. In Fig. 3, images 1 − 8 are representatives of 20 scans (40 μm) steps from the surface to the deep depth of the LC. Image 3 is located on the anterior surface of the LC (ASLC). Laminar pores could not be identified in the fundus photograph, although they could be identified using SLO, but they were obscure. In contrast, multiple laminar pores were clearly identified at each level of the LC images using our OCT. We were also able to show that both the size and shape of laminar pores changed within the LC.

## In vivo visualization of human multiple lamina sheets

**Fig. 4:**
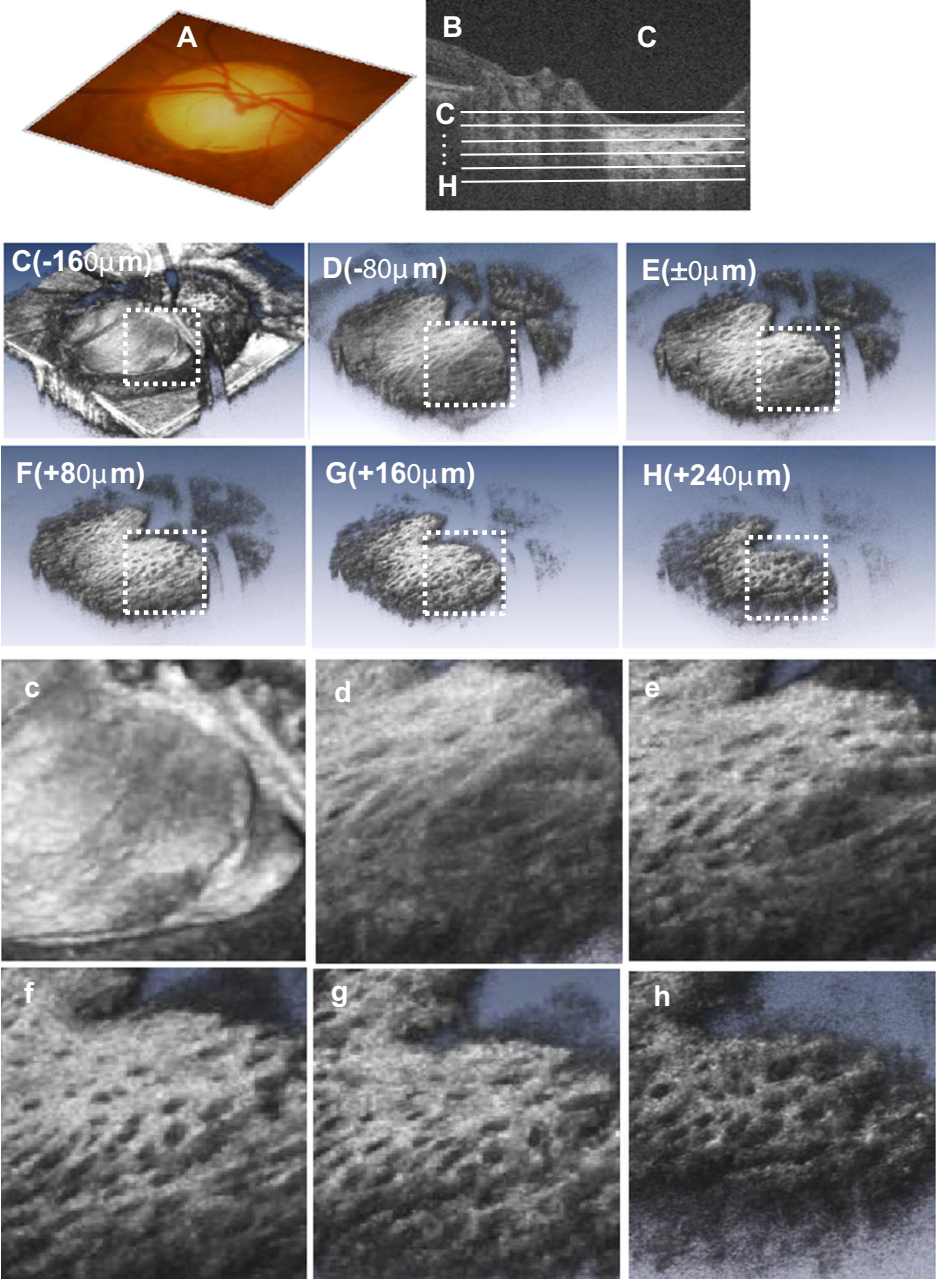
Three-dimensional (3D) OCT imaging of the right lamina cribrosa in a 60-year-old healthy male. **a**, Color disc photograph. **b**, A 3D volume-rendered image of the optic nerve. **c**-**h**, Three-dimensional images of the laminar structure at various levels (in 80 μm steps). En face cross-sectional images at the levels indicated by solid lines in **b**. Nasally, the plate structure is unclear, because of a vascular shadowing effect. **c**-**h**, Magnified images of the same patch of lamina denoted by white dot squares in images **c**-**h**.

Figure 4 shows 3D images of the LC structure at various levels in the normal eye of a 60-year-old patient. These 3D images were accumulated from en face images, using up to 250 scans in 2.0 μm steps. Every 40 scans (80 μm) of the surface, images were removed. Image E is located on the ASLC. Multiple laminar sheet structures were identified in these 3D images, down to a depth of 240 μm from the ASLC. Videos of these structures as moving images are available in supplemental file 2. Overall, laminar sheet structure images could be reconstructed in 41 (89.1 %) eyes. In the other five eyes, laminar pores could be identified, but a 3D sheet image could not be reconstructed because of a small disc, vessel shadow, or tilted disc.

## Comparison of en face images

**Fig. 5:**
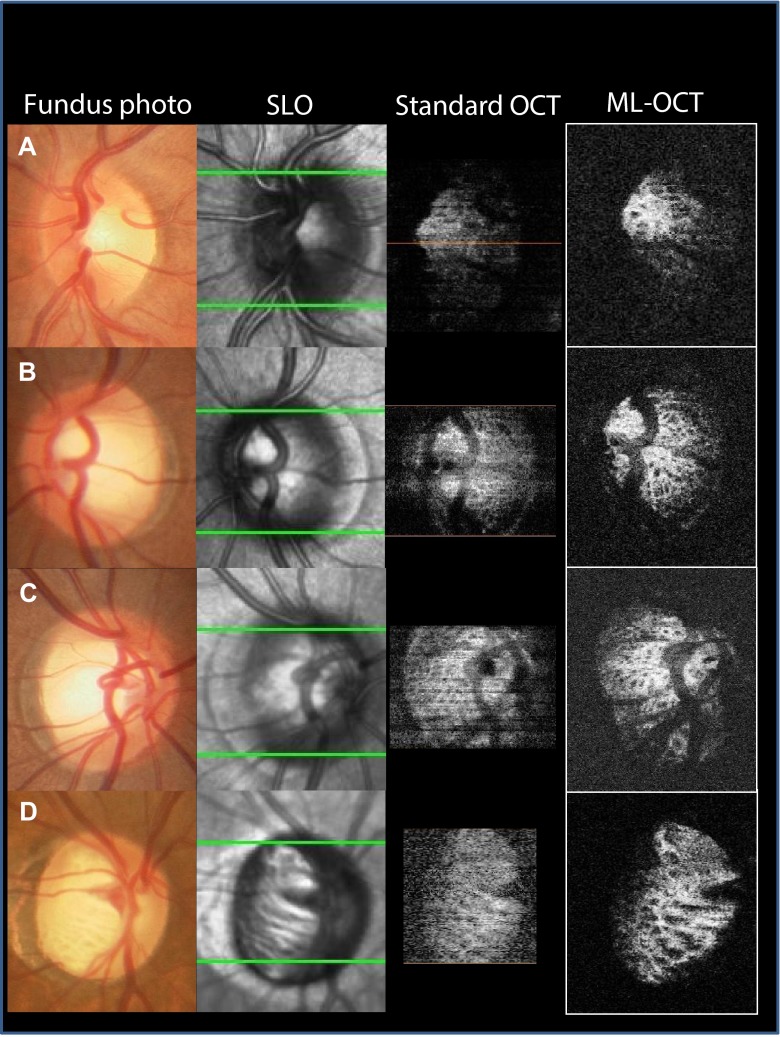
Comparison between fundus photography, SLO, en face imaging from standard OCT, and our OCT. **a**, Images are from the left eye of a 30-year-old male without glaucoma. The refractive error was −3.25D. The lamina pores could not identified from the fundus photograph and SLO image, and were obscured in the standard OCT image. Lamina pores were identified only using our OCT image. **b**, **c**, Images from the left eye of a 36-year-old male without glaucoma (**b**), and the right eye of a 25-year-old male without glaucoma (**c**). The lamina pores were not identified from the fundus photograph, and partially identified from the SLO and standard OCT images. En face image from our OCT clearly identified these pores. **d**. Images are from the right eye of a 70-year-old female with glaucoma. The refractive error was +1.25D. Although slit-like lamina pores were identified in all images, round pores were identified only from our OCT images

Figure 5 shows a disc image using fundus photography and SLO, and thin slice, en face images of an optic disc using standard OCT and our OCT. Although lamina pores were identified in both non-glaucomatous patients and glaucomatous patients using both standard OCT and our OCT, disc images using our OCT more clearly identified lamina pores. Table 2 lists the proportion of images, which were able to identify laminar pores. In 44 of 46 eyes (95.7 %), clear lamina pores using our OCT were identified, whereas 6 (13.0 %), 14 (30.4 %), and 24 (52.2 %) eyes were confirmed using fundus photographs, SLO, or standard OCT, respectively. Taken together, the proportion was significantly larger using our OCT than with the other techniques (*p* < 0.001, McNemar’s test).

**Table 2:** Proportion of images to identify laminar pores

eyes (%)	Fundus camera	SLO	Standard OCT	ML-OCT	P value
Identified clear lamina pores					
Total (%)	6 (13.0)	14 (30.4)	24 (52.2)	44 (95.7)	<0.001*
P value	<0.001†	<0.001†	<0.001†	<0.001†	
glaucoma (%)	5 (50.0)	8 (80.0)	8 (80.0)	10(100)	0.029*
P value	0.063†	0.500†	0.500†	n.a.	
no glaucoma(%)	1(2.8)	6(16.7)	16(44.4)	34(94.4)	<0.001*
P value	<0.001†	<0.001†	<0.001†	n.a.	
P value for glaucoma vs. no glaucoma	<0.001‡	<0.001‡	0.073‡	1.000‡	
Identified lamina pores (including obscure pores)					
Total (%)	14(30.4)	21(45.7)	32(69.6)	46(100)	<0.001*
P value					
glaucoma (%)	7(70.0)	8(80.0)	9 (90.0)	10(100)	0.112*
P value	0.250†	0.500†	1.000†	n.a.	
no glaucoma(%)	7(19.4)	13(36.1)	23(63.9)	36(100)	<0.001*
P value	<0.001†	<0.001†	<0.001†	n.a.	
P value for glaucoma vs. no glaucoma	<0.001‡	0.028‡	0.143‡	1.000‡	

Abbreviations: SLO, scanning laser ophthalmoscopy; OCT, optical coherent tomography; ML, mode-locked (our OCT); n.a., not applicable

*Cochran's Q test, †McNemar's test compared with ML-OCT values

‡Fisher exact probability test compared between glaucoma and no glaucoma eyes

ML-OCT enabled almost all eyes to identify lamina pores , whereas the proportion of images to identify lamina pores were significantly worse in no glaucoma eyes than in glaucoma eyes using both fundus photo, SLO, and standard OCT.

## Discussion

Using our enhanced OCT, we successfully demonstrated the following: 1) clear visualization of in vivo en face images in 2.0 μm steps from the surface to the deep layer within the LC, 2) identifiable laminar pores within the LC in almost all eyes, and 3) clear visualization of 3D images of laminar beams and laminar sheets structures in vivo.

There have been relatively few reports that have shown 3D laminar sheets in human eyes, in vivo, although cross-sectional images of the LC have been reported using commercial OCT. Visualization of these reticular laminar beams using our OCT was consistent with previous histological studies [3]. The strength of our image technology was that it used a broad wavelength laser beam as the light source. Compared with the wavelength range of 60–100 nm using SLD as a light source in current commercial SD-OCT instruments, our OCT used a 200 nm homogeneous wavelength range with an ML, femtosecond laser light source. The theoretical advantage of a ML laser compared with SLD as an OCT light source has been already reported [15]. The axial (longitudinal) image resolution of OCT is determined by the temporal coherence time of the light source, which is inversely proportional to the bandwidth (Δλ), and is dependent on the wavelength width. The axial resolution can be expressed as ΔL =2ln(2)λ^2^ /(πΔλ), where Δλ is the bandwidth and λ is the central wavelength of the light source. To improve axial resolution, homogeneous and broad bandwidth light sources are required. Principally owing to the limited spectral bandwidth of the light source, the spatial resolution of commercial SD-OCT instruments using SLD is limited to 6 μm. Furthermore, this requirement can only be applied if the spectrum of the light source has a Gaussian profile. If there is fluctuation between each mode of the light source, and the phase between the modes is not fixed, the relationship of the Fourier conjugation cannot be maintained between the time intensity profile and the spectral shape of the optical pulse. For the ML laser, an uncertain relationship exists between the spectral width Δν and the pulse width Δt. However, for an SLD, a free running laser, or a Q-switched laser source, such a relationship is not necessarily relevant. We had already described a prototype OCT using a ML laser as light source, with a high resolution of less than 2.0 μm in human eyes [15]. The results of the present study are an extension of our earlier report.

En face images through the deep ONH facilitated visualization of the 3D lamina pores and beams in the lamina cribrosa, in vivo. The distribution, size, and shape of pores within the LC were more readily visible using en face images, compared with optic disc photographs or when using SLO, suggesting that visualization of pores located beneath the nerve fiber tissue was feasible with our instrument.

The LC is a porous connective tissue though which retinal ganglion cell axon bundles pass in transit to the orbital portion of the optic nerve. Healey and Mitchell reported in The Blue Mountains Eye Study that LC pores were visible in 70.8 % of subjects with open-angle glaucoma, but were visible in only 29.3 % of normal eyes [18], concluding that LC pores were less commonly visible in normal eyes using stereo disc photographs. We confirmed that in seven eyes (70.0 %) with glaucoma and only seven eyes (19.4 %) with no glaucoma were pores identifiable using fundus photography, which was comparable to this previous report. In contrast, laminar pores could be identified in all subjects using our OCT. These findings confirm that all normal eyes had laminar pores that were not visible using fundus photography, possibly because of the thick prelaminar tissue and abundant nerve fibers above the LC.

The 3D in vivo observation of laminar tissue is critical because the LC has been suggested to be of central importance in many optic neuropathies such as glaucoma [2, 19], papilloedema [20, 21], and anterior ischemic optic neuropathies [18]. Although SLO, including adaptive optics SLO (AO-SLO), has been superior to fundus photography in visualization of the laminar pores on the surface of the laminar cribrosa [22–24], SLO imaging was limited in its ability to accumulate thin slice images and visualize the entire laminar structure in three dimensions [24]. Consequently, these instruments have difficulty in imaging the deep layers of the lamina cribrosa, and subsequent difficulty in reconstructing detailed 3D images of the LC. More recently, swept-source OCT (SS-OCT), with a 1050 nm tunable laser, allowed 3D, high- penetration imaging of the deep fundus tissue. Takayama et al. successfully used this instrument to discover a local LC defect [25]. Even though they could construct a cross-sectional en face LC image, it was difficult to visualize the 3D lamina sheet imaging, possibly owing to their lower axial resolution (8 μm compared with 2 μm in our instrument).

In the present study, the deeper and more peripheral lamina sheets had fewer laminar beams, more pores, and larger laminar pores. These findings were consistent with previous histological results [26]. In addition, changes in the size and shape of the surface of laminar pores with progression of glaucoma have also been reported [3, 5]. Our OCT enabled us to visualize, in vivo, multiple thin slice, laminar sheet structural images in human eyes. These results are important, because many histological and pathological studies have reported structural differences of the LC between normal and glaucomatous patients [5, 6].

The LC is known to have a sheet structure of many laminar beams. Lamina connective tissues and beams at the LC vary regionally in normal eyes, which likely influence the biomechanical behavior of the LC. Thus, improved visualization of the laminar structure will be valuable in the improved clinical assessment of laminar abnormalities associated with glaucoma. The deformed pore shape and relocation of pores would imply that the nerve fiber bundles passing through these pores are also deformed, which might correlate with vulnerability of the LC to chemical and/or mechanical damages resulting in glaucomatous optic neuropathy. Further studies are, therefore, needed to clarify these possibilities.

### Limitations

This is a pilot study with a small number of subjects, which may not be adequate to obtain statistically significant results in correlation analyses. Thus, further study using more cases will be necessary. Furthermore, retinal trunk vessels often shift to the optic disc and, consequently, prominent shadowing effects by the assembling large retinal vessels may cause poor visualization of the LC. Thus, we could not compare the other parameters such as disc size, area of peri-papillary atrophy, and could not visualize all areas of the LC, especially those overshadowed by vessels. It was also often difficult to determine the posterior borders of LC in this study. Recent reports have determined the borders using averaging scan image and the EDI method [27, 28]. Averaging images might be able to reduce speckle noise, and emphasize the histological borderline. In this study, we obtained single raster scan images, which may explain why the posterior border of the LC was unclear. However, identification of the posterior border of the LC using OCT is still controversial. Mari et al. [29] reported that many studies identified the posterior limit of the signal, and assumed it to be the posterior surface LC, although this assumption was made without any direct comparison to human histology.

In the present study, the deep LC (or retro lamina region) was observed more than 240 μm beneath the ASLC in some subjects (Fig. 4), which was deeper than previously reported using commercial OCT [27, 30]. However, further investigations will be needed to assess fully the extent of the LC thickness.

In conclusion, our OCT enabled us, for the first time, to observe clearly , in vivo, deep LC structure. Based upon these results, use of this instrument should provide researchers with the opportunity to more thoroughly investigate many other optic neuropathies.

## Electronic supplementary material

Below is the link to the electronic supplementary material.

Video 1 Moving image of the optic nerve head raster B-scans and reconstructed en face images. (MPG 10100 kb)

Video 2 In vivo moving images of the lamina cribrosa at various levels. (MPG 3096 kb)
